# Association between Family Structure, Parental Smoking, Friends Who Smoke, and Smoking Behavior in Adolescents with Asthma

**DOI:** 10.1100/tsw.2010.10

**Published:** 2010-01-08

**Authors:** Francisco Vázquez-Nava, José María Peinado-Herreros, Atenógenes H. Saldívar-Gonzalez, Ma Del Carmen Barrientos-Gómez, Francisco J. Beltrán-Guzmán, Jesus Perez-Martin, José A. Cordóva-Fernández, Carlos F. Vázquez-Rodríguez

**Affiliations:** ^1^ Department of Research in Clinical Epidemiology, No. 6 General Hospital of the Mexican Institute of Social Security, Tampico-Madero City, Mexico; ^2^ Department of Research, Faculty of Medicine, Autonomous University of Tamaulipas, Tampico, Mexico

**Keywords:** asthma, smoking, nonintact family

## Abstract

Recent investigations show that the smoking prevalence among asthmatic adolescents is higher than among healthy adolescents, and the causes that lead these asthmatic adolescents to smoke are unclear. We investigated the association between family structure, parental smoking, smoking friends, and smoking in asthmatic adolescents (n = 6,487). After adjusting for sex and age, logistic regression analyses showed that nonintact family structure, parental smoking, and smoking friends are associated with smoking in adolescents with and without asthma. Asthmatic adolescents who reside in the household of a nonintact family have a 1.90 times greater risk of smoking compared with those who live with both biological parents. It is important that parents who have children with asthma be made aware that the presence of smokers in the home and adolescent fraternization with smoking friends not only favor the worsening of asthma, but also induce the habit of smoking.

## INTRODUCTION

Smoking constitutes an important health problem worldwide and has been related to the development of some chronic diseases, including asthma[1,2,3,4,5,6]. Asthma is the most frequent chronic respiratory disease of our time. During its chronic evolution, it can cause exacerbation and aggravation of asthma symptoms, which are favored by the hypersensitivity of the airways to different environmental stimuli, among which air pollution, genetic factors, and inhalation of tobacco smoke stand out[7,8,9,10].

Despite the evidence that shows that inhaling tobacco smoke is associated with the development and aggravation of asthma, recent investigations demonstrate that the smoking prevalence among adolescents is higher in those who have asthma compared with healthy adolescents[3,4,6,11,12]. Even more so, there are reports that show that smoking among asthmatic adolescents continues to increase, and that the causes that lead these young people to smoke are unclear[11].

Several theories have been examined in an attempt to identify the factors that influence smoking behavior in adolescents; no consensus has been reached[13,14,15,16,17,18,19,20,21]. One attractive hypothesis is that the absence of one or both biological parents, or the presence of smokers, usually parents or friends who smoke, within the environment of adolescents can be responsible for the development of smoking behavior[21,22,23]; however, the role of family structure on smoking in asthmatic adolescents has not been studied.

It is clear that nonintact family and the presence of smokers in the environment of adolescents can exert different effects on the development of their behavior, possibly favoring the acquisition of certain habits or risk behaviors, particularly smoking[13,16,21,24,25]. One priority for control of smoking is to identify, but above all to understand, the degree to which familial and social circumstances influence the development of certain risk behaviors in asthmatic adolescents. Accordingly, this study was designed to determine the association between family structure, smoking parents, and smoking friends with smoking in asthmatic adolescents.

## MATERIALS AND METHODS

This is a cross-sectional study based on a population research. A total of 7,000 adolescents who participated in a broader, prior longitudinal study initiated in June 2005, with the primary objective of detecting and preventing addictions, were invited to participate. Of these, 513 adolescents were not included in the final sample due to their not completing the questionnaire. Thus, we collected data on 6,487 adolescents who ranged in age from 13 to 18 years. The sample corresponds approximately to 30% of adolescents attending secondary and preparatory schools in our region. The study was carried out in December 2006 in the Tampico-Madero area of Mexico. This two-city region lies in the southern part of the state of Tamaulipas on the coastline of the Gulf of Mexico, located 542 km northwest of Mexico City. It has an area of 1,492.7 $km^2$ and a population of 605,431 inhabitants.

Permission was obtained for conducting the project from the corresponding educative authorities in the zone. A list of all enrolled students was requested from each of the selected schools. Schools and students were selected by the random numbers system. Questionnaire application was performed in the classroom with the presence of the teacher and with the collaboration of specially trained consulting personnel. Training of questionnaire surveyors was carried out by means of presentations and workshops to establish common criteria regarding the understanding of certain questionnaire items, such as those related to asthma and smoking. In addition, informed consent was obtained from all adolescents and their parents or guardians prior to study participation. Students had the right at all times to withdraw from the study, even after completing the questionnaire.

### Information Gathering

We used the questionnaire developed by the International Study of Asthma and Allergic Diseases in Childhood (ISAAC). The ISAAC program was developed to afford greater validity to epidemiological studies on asthma, allergic rhinoconjuntivitis, and atopic eczema[26,27]. To determine the level of understanding of questionnaire items, we conducted two pilot studies performed with a 15-day difference between them. In the questionnaire, we included general questions concerning life habits and sociodemographic data. Questions were closed, multiple choice, and binomial.

## Measurements of Interest and Coding of Variables

### *Current Asthma*

To establish asthma diagnosis, we included the following questions:

1. Have you ever had wheezing or whistling in the chest at any time in the past?
2. Have you had wheezing or whistling in the chest in the last 12 months?
3. In the last 12 months, have you had a dry cough at night in addition to or apart from cough associated with a cold or a chest infection?

The presence of wheezing and dry cough at night during the last 12 months, and a history of wheezing in the past, was considered evidence of current asthma. This variable was dichotomized as 1 = asthma present and 0 = asthma absent.

Additionally, adolescents were questioned concerning whether they had been diagnosed with asthma by some physician and which type of medications they were using. We sought medications administered for asthma and classified these as inhaled and oral steroids, bronchodilators, or other medications. Information on the drug consumption for asthma was not considered to establish asthma diagnosis.

### *Family Structure*

This variable was defined based on the presence of the biological father, the biological mother, or both biological parents in the adolescent's home. Response options included: (1) both biological parents, (2) biological father and stepmother, (3) biological mother and stepfather, (4) a sole biological parent, (5) one biological parent and other relatives, and (6) no biological parents. This variable was coded as 1 = nonintact family (absence in the home of one or both biological parents) and 0 = intact family (presence in home of two biological parents).

### *Parental Smoking*

The presence of parents who smoked was documented by the response to the question: Which of your parents smokes at home? The options were (1) my father smokes, (2) my mother smokes, (3) both of my parents smoke, and (4) neither of my parents smoke. Parental smoking was considered present when at least one of the adolescent's parents smoked. This independent variable was coded as 1 = smoking parents and 0 = nonsmoking parents.

### *Smoking Friends*

Smoking friends was documented by the response to the question: How many of your best friends smoke? Response options included the following: (1) none of them, (2) some of them, (3) the majority of them, and (4) all of them. We consider the presence of smoking friends when the adolescents responded affirmatively to any of the 2, 3, and 4 response options. This variable was dichotomized as 1= smoking friends and 0 = nonsmoking friends.

### *Active Smoking*

Measurement of this variable was obtained from participant response to the following:

A. Do you smoke? Options for responding were (1) yes, at least one cigarette daily; (2) yes, but less than one cigarette a week; (3) yes, at least one cigarette per month; (4) yes, but only on special occasions; (5) no, I do not smoke.
B. Have you smoked previously? Options were (1) no, I have never smoked, (2) yes, I have smoked one cigarette just to try it, but I have not smoked in the last 6 months; (3) yes, but I quit >12 months ago.

Based on the definition used in National Addiction Survey Mexico 2002[28], we considered adolescents as active smokers when they reported themselves to be smokers at the time of the interview. This dependent variable was coded as 1 = smoker and 0 = nonsmoker.

## Statistical Analysis

Prevalence of smoking, nonintact family, parental smoking, and smoking friends were determined to compare adolescents with and without asthma. We employed the binomial test to identify the differences in proportions of dichotomous variables. Logistic regression models were performed to study the association between nonintact family, parental smoking, smoking friends, and the outcome variable (smoking in asthmatic adolescents, coded as 1 = smoking asthmatic adolescents and 0 = nonsmoking asthmatic adolescents). Adjusted odds ratios (OR) and their 95% confidence intervals (CI) were calculated. In evaluating the association, we adjusted for the following confounders: gender and age. Data were analyzed by means of the SPSS 10.0 statistical package software. Any value of $p < 0.05$ was considered significant.

The study was approved by the Ethics Committee of the No. 6 Regional General Hospital of the Mexican Institute of Social Security, and the Faculty of Medicine of the Autonomous University of Tamaulipas, Tampico

## RESULTS

Current asthma prevalence was 10.6% and active smoking was 16.5%. A nonintact family was reported by 23.5% of adolescents and 20.3% reported having smoking friends. Table 1 shows the percentages of gender, age, family structure, active smoking, parental smoking, and smoking friends in adolescents with and without asthma. According to binomial analysis, the percentage of active smoking (19.4 vs. 16.0%), smoking parents (34.2 vs. 30.3%), and smoking friends (23.0 vs. 20.0%) was greater in the group of asthmatic adolescents compared with nonasthmatic adolescents.

A higher percentage of asthmatic adolescents who smoke (Table 2) was found in youngsters ≥15 years of age (25.5 vs. 16.1%), who resided in a nonintact family (27.3 vs. 16.3%), who had smoking parents (27.8 vs. 15.1%), and who had friends that smoke (42.3 vs. 12.7%), compared with those asthmatic adolescents ≤15 years of age, who reside with a intact family, and who do not have parents and friends that smoke. Results of the logistic regression models show that after controlling for sex and age, a nonintact family (adjusted OR = 1.90; 95% CI = 1.23–2.91), smoking parents (adjusted OR = 1.71; 95% CI = 1.13–2.59), and smoking friends (adjusted OR = 4.55; 95% CI = 2.98–6.95) are associated with the habit of smoking in asthmatic adolescents.

In the group of adolescents without asthma (Table 2), we found a higher percentage of smoking adolescents in those ≥15 years of age (20.4 vs. 13.7%), who reside in nonintact families household (23.5 vs. 14.2%), and who have parents (25.1 vs. 12.7%) and friends (39.5 vs. 10.7%) who smoke, compared with adolescents ≤15 years of age, who reside with a intact family, and who do have not parents and friends that smoke. Logistic regression analysis showed that after controlling for sex and age, nonintact family (adjusted OR = 1.79; 95% CI = 1.52–2.12), parental smoking (adjusted OR = 2.01; 95% CI = 1.73–2.35), and smoking friends (adjusted OR = 4.77; 95% CI = 4.09–5.57) are associated with smoking in nonasthmatic adolescents.

**TABLE 1**
**Percentages of Gender, Age, Family Structure, Active Smoking, Parental Smoking, and Smoking Friends among Adolescents With and Without Asthma (6,487 Adolescents)**

| | Asthmatics (n = 685) | | Nonasthmatics (n = 5,802) | | *p* Value* |
|---|---|---|---|---|---|
| | N | % | N | % | |
| Boys | 292 | 43.0 | 3,107 | 54.0 | 0.000 |
| Girls | 393 | 57.0 | 2,695 | 46.0 | 0.000 |
| Age (years) | | | | | |
| ≤15 | 446 | 65.0 | 3,635 | 63.0 | 0.000 |
| ≥15 | 239 | 35.0 | 2,167 | 37.0 | 0.000 |
| Intact family | 491 | 71.7 | 4,474 | 77.1 | 0.000 |
| Nonintact family | 194 | 28.3 | 1,328 | 22.9 | 0.000 |
| Active smoking | 133 | 19.4 | 939 | 16.0 | 0.000 |
| Parental smoking | 234 | 34.2 | 1,757 | 30.3 | 0.000 |
| Father who smokes | 135 | 19.7 | 1,055 | 18.2 | 0.000 |
| Mother who smokes | 44 | 6.4 | 323 | 5.6 | 0.000 |
| Both parents smoke | 55 | 8.0 | 379 | 6.5 | 0.000 |
| Smoking friends | 156 | 23.0 | 1,159 | 20.0 | 0.000 |

* Binomial test.

**TABLE 2**
**Effect of Gender, Age, Family Structure, Parental Smoking, and Smoking Friends on Smoking among Adolescents With and Without Asthma**

| | Asthmatic (n = 685) | | | | | Nonasthmatics (n = 5802) | | | | |
|---|---|---|---|---|---|---|---|---|---|---|
| | Yes | % | No | % | Adjusted OR (95% CI)[a] | Yes | % | No | % | Adjusted OR (95% CI)[a] |
| Male | 59 | 20.2 | 233 | 79.8 | 1.11 (0.73–1.68)** | 502 | 18.6 | 2,193 | 81.4 | 1.40 (1.20–1.63)* |
| Female[b] | 74 | 18.8 | 319 | 81.2 | | 437 | 14.1 | 2,670 | 85.9 | |
| ≥15 years | 61 | 25.5 | 178 | 74.5 | 1.44 (0.95–2.19)** | 442 | 20.4 | 1,725 | 79.6 | 1.46 (1.26–1.70)* |
| ≤15 years[b] | 72 | 16.1 | 374 | 83.9 | | 497 | 13.7 | 3,138 | 86.3 | |
| Nonintact family | 53 | 27.3 | 141 | 72.7 | 1.90 (1.23–2.91)* | 305 | 23.5 | 1,023 | 77.0 | 1.79 (1.52–2.12)* |
| Intact family[b] | 80 | 16.3 | 411 | 83.7 | | 634 | 14.2 | 3,840 | 85.8 | |
| Parental smoking (+) | 65 | 27.8 | 169 | 72.2 | 1.71 (1.13–2.59)* | 500 | 25.1 | 1,491 | 74.9 | 2.01 (1.73–2.35)* |
| Parental smoking (–)[b] | 68 | 15.1 | 383 | 84.9 | | 572 | 12.7 | 3,924 | 87.3 | |
| Smoking friends (+) | 66 | 42.3 | 90 | 57.7 | 4.55 (2.98–6.95)* | 519 | 39.5 | 796 | 60.5 | 4.77 (4.09–5.57)* |
| Smoking friends (–)[b] | 67 | 12.7 | 462 | 87.3 | | 553 | 10.7 | 4,619 | 89.3 | |

*Note:* (+) indicates characteristic present; (–) indicates characteristic absent; 95% CI, 95% confidence interval; OR, odds ratio.

[a] Adjusted for sex and age.

[b] Reference category.

* $p < 0.05$.

** $p > 0.05$.

## DISCUSSION

Our results show that living in a nonintact family household, the presence of smoking parents in the home, and the presence of smoking friends appear to be associated with smoking among asthmatic adolescents.

Past research has shown that smoking rates among asthmatic adolescents are higher than those found in nonasthmatic adolescents[4]. The results of this study agree with those authors. We found that the percentage of smoking is higher in asthmatic adolescents than in nonasthmatic adolescents. Our results suggest that preventive smoking programs in general are not functioning adequately, and that these require review and reinforcement of their strategies to sensitize the population to the harm caused to health by smoking, acting mainly in groups of individuals who are carriers of respiratory diseases such as asthma.

According to previous reports, the number of single-parent families has increased in the last years[29,30]. Living in a household with one, or no, biological parent has been associated with the development of risk habits and behaviors in adolescents[14,16,24]. According to the analysis, asthmatic adolescents who live in nonintact family households have a 1.9 times greater risk of smoking compared with those residing with both biological parents. To our knowledge, this is the first investigative work that shows the effect of living in a nonintact family household on the smoking habits of asthmatic adolescents. It is probable that this effect is due to a more tolerant attitude toward smoking by the permanent parent, or even to the unaware and absence of control of parents concerning the adolescent's activities outside the home[24,31,32].

Based on the results, the presence of smoking parents favors the development of smoking in asthmatic adolescents. Previous studies agree with this association.[21]. Otten et al. found that an asthmatic adolescent who smokes is 4.25 times more likely to have parents who smoke, compared with adolescents who do not smoke[33]. The mechanisms that determine the relationship between parents who smoke and smoking in asthmatic adolescents are unclear. During adolescent development, the changes that present during this life stage can play a relevant role in the development of certain habits, such as smoking; these changes can present at younger ages and with greater frequency when these are based on direct experience. The presence of smoking parents in the homes of asthmatic adolescents can be perceived by these young people as a sign that their disease is not severe, that smoking is not harmful to health, or as approval of smoking.

According to the analyses in this study, asthmatic adolescents who socialize with smoking friends have a four times greater risk of smoking compared with asthmatic adolescents with nonsmoking friends. This effect has been reported previously by Zbikowski et al., who on studying risk factors for smoking among adolescents, found that asthmatic adolescents who fraternize with smoking friends had a 8.7 times greater risk of smoking in comparison with those with nonsmoking friends[21]. Some of the following factors can favor this effect. During adolescence, young people are subjected to many pressures to acquire habits that are harmful to health. In addition, at this age, adolescents spend more time with their friends than with their families, and the opinion of friends in relation to the acquisition of certain risky habits and behaviors can be more influential than that of their parents.

Some investigators have found that the risk of smoking in asthmatic adolescents increases with age and feminine gender[16]. In this study, our group did not find that gender and age are associated with smoking in asthmatic adolescents.

This study possesses certain limitations that should be taken into consideration. Information generated by this study arises from a population study. Data correspond to adolescents who attended secondary or preparatory schools; thus, these results cannot be generalized to open populations. In Mexico, approximately 30% of adolescents aged 13–18 years are not enrolled in any secondary school, preparatory school, or university-level course[30]. The prevalence of smoking found is based on that declared by the study participants; no objective smoking tests, such as measurement of carbon dioxide, were conducted.

Additionally, information regarding asthma in the current study was derived from self-reports of the adolescents themselves using the ISAAC questionnaire. However, several studies have shown that this instrument can be considered effective for measuring diagnosis of asthma in epidemiological studies[27].

In conclusion, the data obtained in this study show that nonintact family structure, the presence of smoking parents, and socializing with smoking friends appear to be important inducers of smoking in asthmatics adolescents. It is fundamental that preventive smoking programs for asthmatic adolescents involve all family members in order for the latter to know that asthma is a serious disease. Also, parents who have children with asthma must be made aware that the presence of smokers in the home and adolescent fraternization with smoking friends not only favor the worsening of asthma, but also induce the habit of smoking.
